# Clinical and cost-effectiveness of ‘Live Well with Parkinson’s’ self-management intervention versus treatment as usual for improving quality of life for people with Parkinson’s: study protocol for a randomised controlled trial

**DOI:** 10.1186/s13063-023-07700-7

**Published:** 2023-12-05

**Authors:** Kate Walters, Megan Armstrong, Benjamin Gardner, Gareth Ambler, Rachael Hunter, Bev Maydon, Nathan Davies, Catherine Atkinson, Richard Brown, Tasmin Rookes, Daniel Davis, Anette Schrag

**Affiliations:** 1 Department of Primary Care and Population Health, University College London, Royal Free Campus, Rowland Hill Street, London, NW3 2PF UK; 2 Department of Psychology, University of Surrey, Guildford, UK; 3 Homerton Healthcare NHS Foundation Trust, London, UK; 4 Institute of Psychiatry, Psychology and Neuroscience, King’s College London, London, UK; 5 MRC Unit for Lifelong Health and Ageing, UCL, London, UK; 6 Institute of Neurology, UCL, London, UK

**Keywords:** Parkinson’s, Self-management, Long-term conditions, Behaviour change, Randomised controlled trial

## Abstract

**Background:**

The Live Well with Parkinson’s Self-Management Toolkit is designed for use in the NHS to support people with Parkinson’s, their carers and health professionals in managing motor and non-motor symptoms and promoting well-being. The Toolkit was developed based on theory-based behaviour change and self-management techniques in consultation with people living with Parkinson’s and health and social care practitioners. There are digital (e-Toolkit) and paper (manual) versions.

**Methods:**

Single-blind two-arm randomised controlled trial RCT of clinical effectiveness and cost-effectiveness of the Toolkit, facilitated by up to six sessions with a trained non-specialist supporter, in improving quality of life. People with Parkinson’s will be assessed at baseline, 6 and 12 months. Assessors will be blind to the treatment group. The primary outcome measure is the Parkinson’s Disease Questionnaire (PDQ-39, Parkinson’s related quality of life) score at 12 months. Secondary outcome measures include the MDS Unified Parkinson’s Disease Rating Scale (Part I, II, III, IV), EQ-5D, and a Client Service Receipt Inventory shortened, adapted for Parkinson’s. Carer outcomes include the Zarit Carer Burden Inventory and Carer Quality of Life Questionnaire for Parkinsonism. A total of 338 people with Parkinson’s, and their carers if appropriate, will be recruited from diverse settings across England. Those with advanced dementia, at end-of-life or with atypical Parkinsonism will be excluded. A parallel mixed methods process evaluation will explore the factors promoting or inhibiting implementation, uptake, use, effectiveness and cost-effectiveness of the Toolkit and sessions.

**Discussion:**

If successful, the Live Well with Parkinson’s Toolkit could be used as a model for other complex long-term disorders, including dementia. This would bridge existing gaps in the NHS (as shown by the national Parkinson’s audit data), by enabling patients and carers to access personalised information, advice and support on symptom management and ‘living well’ with Parkinson’s.

**Trial registration:**

ISRCTN92831552. Registered on 26th Oct 2021.

**Supplementary Information:**

The online version contains supplementary material available at 10.1186/s13063-023-07700-7.

## Background

Parkinson’s disease (PD) is the second most common neurodegenerative disorder, affecting around 127,000 people with the condition in the UK, and one in 50 people over 65 years [1]. The number of people affected and the associated costs are increasing with the ageing population and are predicted to double by 2030 [2]. PD is associated with a complex range of disabling and distressing non-motor symptoms, including dementia and cognitive impairment, apathy, depression/anxiety, psychosis, bowel/bladder dysfunction, fatigue, sleep problems and pain [3]. PD is associated with a 45% increased risk of hospital admission and longer stays than others of the same age [4]. Due to the complexity of PD, treating common symptoms can be difficult for non-specialist health professionals, such as General Practitioners (GPs) [5, 6].

Many aspects of PD can be treated or managed before they become severe problems, if recognised and supported appropriately. Increasingly, patient or carer participation in management (i.e. self-management) is incorporated into health care for long-term conditions (LTCs), as this can allow people to take control and improve outcomes in the face of restricted resources and fragmentation of health care. The current policy direction of the National Health Service (NHS) (e.g. NHS Long-term Plan) emphasises self-management [7]. Interventions to support self-management include education, psychological support, strategies to support treatment adherence and tailored practical support, including liaison with healthcare professionals and other agencies [8]. There is evidence that supported self-management for LTCs can be clinically effective, decreases health care utilisation and does not compromise patient outcomes [9].

Information and advice about Parkinson’s and associated symptoms and treatments are available from charities such as Parkinson’s UK [10], who also support local Self-Management Programme/Peer-support groups. In the US, there are ongoing evaluations of proactive individual management approaches and social self-management in PD [11]. However, there is currently no effective comprehensive, personalised self-management tool designed for use in a national health service to support people with Parkinson’s, their carers and specialist or non-specialist health professionals in managing motor and non-motor aspects of the condition and promoting well-being. A systematic review of self-management in Parkinson’s found mixed evidence for the effectiveness of self-management, with few large-scale, high-quality studies [12]. However, a further evaluation of these interventions with interviews with people with Parkinson’s [13] and Health Care Professionals (HCPs) [14] identified that those interventions did not include key self-management components [15] or the perspectives of people with Parkinson’s.

We have co-designed, with people with Parkinson’s and their carers, a self-management facilitated Toolkit (the ‘Live well with Parkinson’s’ Toolkit). The Toolkit was developed by synthesising systematic reviews, qualitative interviews, co-design workshops, and theories such as self-management [16] and behaviour change theory [17] (www.ucl.ac.uk/pdcare). We then tested the Live Well with Parkinson’s Toolkit in a feasibility study. We recruited 35 (100% of our target) people with Parkinson’s from three secondary care sites in and around London. The study successfully recruited within the expected timeframe, with 31 (89%) participants remaining at 3 months and minimal missing data (full details will be published later). The process evaluation indicated that the intervention was well received according to participant interviews and a survey. The study confirmed the Live Well with Parkinson’s Toolkit was feasible to deliver with high levels of engagement (34 participants [97%] engaging). Given the success of the feasibility study, we aim to test the Live Well with Parkinson’s in a definitive trial.

### Aims

1. Test the clinical effectiveness of Live Well with Parkinson’s in maintaining the quality of life in a randomised controlled trial (RCT) in comparison to treatment as usual (TAU).
2. Determine the cost-effectiveness of Live Well with Parkinson’s comparison to TAU.
3. Explore the context, mechanisms, and impact of the intervention for different populations (age, gender, deprivation, and ethnicity) and barriers and facilitators to implementation at scale.

## Methods

We will carry out a two-arm, single-blind, parallel-group, superiority RCT comparing the intervention group, receiving access to the ‘Live Well with Parkinson’s’ Toolkit to TAU with a 1:1 allocation, including a cost-effectiveness analysis and mixed methods process evaluation. This protocol paper focuses on the main clinical and cost-effectiveness RCT. A detailed protocol for the aligned process evaluation will be reported separately. This protocol has been drafted following the SPIRIT checklist for Trials [18], see Additional file 1.

### Eligibility criteria

The trial will include community-dwelling adults (i.e. aged 18 and above) with a confirmed diagnosis of Parkinson’s Disease (defined using UK Brain Bank Criteria [19]), including those with dementia diagnosed at least 1 year after their Parkinson’s diagnosis. Participants must be able to engage in the intervention and study assessments independently or with the support of a carer or family member.

We will exclude those: with a clinical diagnosis of Atypical Parkinsonism; who are currently an inpatient or living in a care home; with severe cognitive impairment who lack the capacity to take part (MoCA score < 11) [20]; who are unable to engage in the intervention due to visual impairment or language barriers (and no carer or family member to support them to engage); who have a life expectancy < 6 months; or who are participating in another clinical trial/study likely to impact or interfere with the Live Well with Parkinson’s intervention.

### Intervention

The intervention includes TAU plus the manualised Live Well with Parkinson’s intervention. A detailed breakdown of the Live Well with Parkinson’s intervention is provided according to the TIDIER checklist in Additional file 2. Live Well with Parkinson’s includes up to six supporter sessions and access to the ‘Live Well with Parkinson’s’ Toolkit.

#### Toolkit

The Toolkit is based on evidence from a series of systematic reviews [13, 21] and qualitative studies with people with PD and health care professionals (HCP) [14, 22] conducted by the study team. It is based on evidence from effective health promotion interventions and incorporates behaviour change techniques drawn from theories such as the COM-B model [17]. An asset-based approach underpins the overall intervention, focusing on maintaining independence, health and current activities rather than addressing deficits. The Toolkit was co-designed with people with Parkinson’s, carers, health and social care professionals and Parkinson’s disease experts and is available in paper format and online. Both versions can be shared with participants’ carers and HCPs either as a whole toolkit or selected sections. Please see www.ucl.ac.uk/pd-care for more information.

The Toolkit consists of 64 information sections on what Parkinson’s is, symptoms, therapies/treatments, optimising well-being, and practical advice. Each content section has been through review by two members of the team (at least one of whom was clinical), an expert in the specific area and by PPI team members, and subject to ‘readability’ review (aim of Flesch Reading Ease target of 70 +) [23]. The Toolkit also comprises personalised sections titled as follows: (1) About Me (including information on their contacts, support and planning future care); (2) My Health (including information on their health conditions, medication, treatments, and research involvement); (3) Symptom Review (including a list of symptoms they experience and the severity of them); (4) My Well-being (to identify health behaviours they would like to maintain or improve); (5) My Tracker (to track medications, activities, and symptoms allowing participants to identify patterns and specialists to get a better idea of what participants are experiencing); (6) Appointments/calendar (to allow participants to store all their healthcare appointments in one place); and (7) To-do lists/Notes.

#### Intervention supporter sessions

Participants, and, if participants wish, their carers, will receive around four (up to six if needed) sessions over 6 months led by a ‘supporter’. The supporters will be trained professionals with a background in healthcare (e.g. psychology, occupational therapy, nursing), social care or third sector organisations (e.g. care navigation, social prescribing), with some experience working/caring for people with Parkinson’s or other complex long-term conditions. They will receive training to deliver the intervention. The sessions will be around 60–90 min for the first two sessions and around 30 min for the remaining sessions. The aim of the sessions is to encourage participants to self-manage their condition using the ‘Live well with Parkinson’s’ Toolkit. The supporter will follow a manual and checklists covering support navigating the Toolkit, understanding the benefits of using the different sections, assisting in creating well-being priorities (goals) and using behaviour change techniques to help implement priorities long-term. These sessions will be conducted online via videocall, by telephone or face-to-face when appropriate. Supervision and training is detailed in Supplementary Material 1.

### Control

The control will be TAU: usual care from existing sources (GP, Parkinson’s specialist service + / − NHS Parkinson’s Disease Nurse Specialists (PDNS)). TAU in the current NHS is delivered by primary care together in most instances with secondary care (neurology or geriatrics) consultations every 6 to 12 months, with a PDNS where available who provides information, reviews, and a telephone service for queries between appointments [24]. Referrals to other specialties and therapists (physiotherapy, occupational therapy, speech and language therapy, social care services, etc.) are made as appropriate.

### Setting and recruitment

Participants and their carers will be recruited through secondary and primary care; based on previous successful recruitment of people with Parkinson’s through neurology and care of the elderly clinics, PDNSs, and primary care [25]. Sites have been selected to represent teaching hospitals, district general hospitals and primary care across inner city, suburban and rural locations, including in areas with diverse and more deprived populations (GP practices in areas scoring 1–4 on the latest version of the Index of Multiple Deprivation (IMD) scale). Specialist services will include those led by neurologists and geriatricians, and both hospital and community-based PDNSs. The study will collaborate with Parkinson’s UK for recruitment (www.parkinsons.org.uk).

### Internal pilot and progression criteria

The first wave of recruitment for the initial 6 months will form an internal pilot, to further test trial recruitment procedures and participant willingness to be randomised. The stop/go progression criteria at 6 months include a minimum 70% recruitment and randomisation rate; intervention uptake; retention rate and no serious intervention-related adverse events.

### Randomisation and blinding

We will randomise participants 1:1 to receive the Live Well with Parkinson’s intervention or TAU only. Minimisation will be used to perform individual randomisation based on site. Randomisation will be carried out by unblinded staff members using the remote computerised web-based application ‘Sealed Envelope’, provided by UCL’s PRIMENT Clinical Trials Unit (CTU). Participants will be informed of their group allocation by phone call. Participants in the TAU arm will also receive a letter confirming their group allocation and reminding them of their follow-up time points. Outcome assessors, the Chief Investigator, the Trial Manager and Trial Management Group members who are not site Principal Investigators or responsible for intervention delivery will be blinded to participant allocation. Systems and strategies to maintain blinding will be implemented, for example, outcome assessments with an alternative blinded researcher from another site, if a research assistant becomes accidentally unblinded during the study.

### Outcomes

Clinical outcomes will be measured at baseline, 6 months and 12 months, ideally within + / − 2 weeks of this date, by a researcher blind to intervention status (see Table 1). Maintenance of blinding will be documented using a Researcher Perception form. Assessments will be completed face-to-face at a clinic or participant’s home, or remotely (by video or telephone) according to their location and the participant’s preferences. Assessments can be divided into two sessions with some assessments completed by the participant and some with a researcher. Participants will receive a £20 voucher for completing the baseline assessments and £10 for each follow-up completed. Data will be kept confidentially at sites or in Data Safe Haven and entered into a secure web-based database developed for the trial. A monitoring plan is in place to ensure data quality.

**Table 1:** Outcome assessment schedule for people with Parkinson’s and carers

Construct	Outcome measure	Baseline	6 months	12 months
**People with Parkinson’s disease**				
Socio-demographic characteristics		X		
Cognitive impairment	Montreal Cognitive Assessment test Version validated for phone administration [20]	X		
Health status and quality of life	Parkinson’s Disease Questionnaire (PDQ-39) [26]	X	X	X
Non-motor and motor experiences of daily life	Unified Parkinson’s Disease Rating Scale (MDS-UPDRS) [27]	X	X	X
Non-motor symptoms	Non-Motor Rating Scale (MDS-NMS) [28]	X	X	X
Self-efficacy	Self-Efficacy for Managing Chronic Disease (6- Items) [29]	X	X	X
Psychological well-being	General Health Questionnaire (GHQ12—12 items) [30]	X	X	X
Capability to manage health conditions	Patient Activation measure (13 items) [31]	X	X	X
Economic evaluation	Client Service Receipt Inventory-shortened (CSRI), adapted for Parkinson’s [32, 33]	X	X	X
	EQ-5D-5L (5 item VAS) [34]	X	X	X
	ICECAP-O, (5 items) [35]	X	X	X
**Carers**				
Socio-demographic characteristics		X		
Carer burden	Zarit carer burden inventory (22 items) [36]	X	X	X
Quality of life	Carer Quality of Life questionnaire for Parkinsonism (26 items) [37]	X	X	X

The primary outcome is the Parkinson’s Disease Questionnaire (PDQ-39) [26] score at 12 months. PDQ-39 is a valid and reliable measure of quality of life, widely used in Parkinson’s trials. Secondary outcomes and carer outcomes collected are reported in Table 1. Self-efficacy, capability to manage health conditions and carer measures are being collected as part of our aligned process evaluation and will be reported separately.

### Sample size

We aim to recruit a sample of 338 people with Parkinson’s (169 per arm). To detect a 4.7-point difference in our primary outcome PDQ-39 [38] with 90% power and 5% significance using a baseline-adjusted (ANCOVA) analysis, 135 participants per arm are required, assuming a SD of 19.8 and a correlation between baseline and follow-up measurements of 0.8 [27, 39]. Allowing for 20% attrition at 12 months in each arm increases the total to 338 participants. We note that a change of 4.7 points is in line with what is considered the minimal clinically important difference for PDQ-39 [39]. The standard deviation of 19.8 was derived from the PDQ-39-SI document (Tables 7.1 and 7.2) [40] and the assumed correlation of 0.8 between baseline and follow-up measurements was based on results on our secondary outcome MDS-UPRDS [41].

### Process evaluation

A detailed protocol of our process evaluation will be reported separately. In summary, the process evaluation will collect quantitative and qualitative data alongside the main trial. The process evaluation will explore the context, mechanisms and implementation of the intervention, including fidelity, intervention acceptability, and recommendations for improvement. We will also use the trial data to assess reach and the mechanisms that impact the primary outcome.

## Statistical methods

### Main statistical analysis

A comprehensive statistical analysis plan will be developed and agreed with the trial’s oversight committee. Descriptive analysis (e.g. summary statistics, plots) will be performed to summarise baseline characteristics by treatment group and to investigate the distribution of the primary outcome, PDQ-39, across participants. The primary analysis will use a three-level linear mixed model to compare PDQ-39 scores at 12 months between treatment groups, adjusting for baseline PDQ-39, age and socio-economic status as fixed effects, and participant and site as random effects. All analyses will be performed on an intention-to-treat basis and all modelling assumptions will be checked (e.g. using residuals). A Complier Average Causal Effect (CACE) analysis will be performed as a sensitivity analysis to investigate the potential impact of non-compliance. Missing data will be investigated, and multiple imputation used if appropriate. Secondary outcomes will be compared using similar methods to the primary outcome. We will undertake a pre-specified sub-group analysis exploring the effectiveness in early (diagnostic/maintenance) vs. advanced (complications/palliative) Parkinson’s. Participant and carer data will be linked to explore possible associations between carer burden and QoL with participant factors such as disease severity. See Additional file 3 for a full statistical analysis plan.

### Economic evaluation

A health economics analysis plan (HEAP) will be written and signed off by the trial steering committee prior to analysis. The incremental cost per quality-adjusted life year (QALY) gained of the intervention compared to TAU from (i) health/social care perspective and (ii) societal perspective using trial data will be calculated. Additional analyses will calculate the cost per capability adjusted life year (CALY) gained from both cost perspectives. QALYs will be calculated from the EQ-5D-5L and as the area under the curve adjusting for baseline [42]. CALYs will be calculated using the ICECAP-O and the associated index values [43]. Resource use will be costed using nationally published sources (Personal Social Services Research Unit (PSSRU) [44], NHS national cost collection [45] and the British National Formulary (BNF) for medications) [46]. The cost of the intervention including staff training, administration and delivery will be included in the costs of the intervention group. Mean incremental costs, QALYs and CALYs for the intervention compared to the control group will be calculated using linear regression adjusting for baseline, socio-economic fixed effects and site as a random effect. Bootstrapping will be used to calculate 95% confidence intervals, cost-effectiveness acceptability curves (CEAC) and cost-effectiveness planes (CEP). A life-time decision model will be developed to project the lifetime costs and QALYs from a health and social care cost perspective to extrapolate the results of the trial. The model will be based on a previously developed model of Parkinson’s progression and associated costs and QALYS [47]. Probabilistic sensitivity analysis will be used to construct CEACs and CEPs.

### Process evaluation analysis

We will undertake a thematic analysis of our qualitative data alongside supplementary analyses to explore hypothesised mechanisms for intervention effects and to evaluate reach, dose and intervention fidelity as part of our process evaluation. This will be described in detail in a separate protocol.

### Patient and public involvement (PPI)

This project has a study-specific PPI Advisory Panel of eight people with experience of Parkinson’s and carers of people with Parkinson’s, acting as a consultative and advisory forum for all stages of the study, meeting regularly throughout the study and feeding back to the Steering Committee and study team. The PPI panel have taken an active role through our co-design process in developing the intervention itself, specifically in selecting and reviewing content topics, design, and aspects of supporter role. The group will provide mutual support, co-facilitated by the research team (trial manager) and the PPI Lead (BM). The views of the PPI advisory panel will be integrated throughout.

### Oversight and monitoring

Live Well with Parkinson’s RCT has an independent Trial Steering Committee which also acts as the Data Monitoring and Ethics Committee. This committee meets twice yearly to review the trial and make recommendations. PRIMENT CTU oversee the trial auditing, which will be carried out regularly with the principal investigators of the sites and trial manager.

### Changes to the protocol

Any changes to the protocol we will notify the sponsor, PRIMENT CTU, and funder first and following approvals we will notify the study sites if needed. The revised protocol will be sent to the PI to add the investigator site file. The trial manager will add the updated protocol to the trial management file and clinical trial registry. Any deviations from the protocol will be documented using a breach report form and sent to PRIMENT CTU to review.

### Ethics

The study was approved by the London Queen Square Research Ethics Committee (REC (21/LO/0562)). Researchers will seek audio-recorded informed verbal consent or e-consent if remote or informed written consent if face-to-face from all participants after being sent an information sheet and given the opportunity to ask questions to the researcher (See Additional file 4). Researchers will be trained in Good Clinical Practice and the Mental Capacity Act 2005. Any concerns about participants’ welfare will be discussed with KW (practising GP) or AS (practising neurologist) or intervention supervisors and appropriate local services informed with the participant’s consent where possible. All serious adverse events (SAEs) will be reviewed by KW or AS who will complete the sponsor’s SAE form and send this to PRIMENT CTU within 24 h of becoming aware of the event. Where the SAE is unexpected and thought to be related to the procedure this will be reported to the REC by PRIMENT CTU within 15 days of becoming aware of the event.

### Trial status

Recruitment began in January 2022 and finished in July 2023, with the last follow-up being in July 2024. The latest protocol is Version 1.4 (23rd March 2023).

### Dissemination

On completion of the study, the data will be analysed and tabulated and a Final Study Report prepared. The full study report will be accessible on the UK Clinical Trial Network. The dissemination strategy will include the following:

- *Engagement with stakeholders:* A symposium to present findings on completion; policy briefing documents/individual engagement with key policy makers from the Department of Health, NHS England, interest groups (e.g. Parkinson’s UK); regional engagement with NHS Local Area Teams, Clinical Commissioning Groups (CCGs) and Local Authorities targeting Integrated Care leads.
- *Commissioning Live well with Parkinson’s:* A costing model will be developed to commission training and access longer term. Throughout the duration of the programme grant work will take place with the third sector, NIHR Head of Impact and NHS England to develop appropriate commissioning models and long-term sustainability of the intervention.
- *Academic dissemination:* Traditional methods of academic dissemination and social media will be used for peer-reviewed academic publications. Findings will be presented at key relevant national and international conferences and disseminate our findings/publicise our papers via social media (e.g. via ResearchGate project, Twitter).
- *Public dissemination:* Close work with the study PPI advisors and Parkinson’s UK will take place to implement a comprehensive public dissemination strategy. Social and print media will be used, and dissemination of results through partnership with Parkinson’s UK and presentations at patient/carer fora. PPI members will have an active role, leading on some aspects, co-author papers and be acknowledged, as appropriate.
- *Participants:* We plan to notify the participants of the outcome of the study, with access to the publication, and we will present the study locally for staff and the public, which participants will be invited to. Participants will be able to specifically request results from the PI and this information will be provided at the next consultation or in a letter depending on the participant’s preference. At the end of the study, participants will be provided with a summary of the findings from the programme, written in plain English with input from our public advisory panel. More detailed information will be made available via our study website, with hard copies sent to those participants requesting them.

## Discussion

This RCT will evaluate the clinical and cost-effectiveness of a facilitated self-management intervention aiming to promote quality of life and enable people to ‘live well’ with Parkinson’s. Live Well with Parkinson’s is a novel web-based digital intervention with a manualised paper version to maximise reach, that includes multiple domains (e.g. information, symptom and medication tracking, goal development) and integrates behaviour change and maintenance. It has a theoretical basis and rigorous co-design development process in partnership with people with lived experience of Parkinson’s alongside health and social care practitioners and experts.

The trial will recruit from diverse sites across England, aiming to include a representative population of people living with Parkinson’s. It will provide important evidence regarding the effectiveness and cost-effectiveness of a facilitated digital self-management intervention for a complex long-term condition, which will also be relevant for other similar more complex conditions. A parallel process evaluation will explore potential mechanisms of effect and factors that influence implementation across routine health settings.

This is a pragmatic trial, aiming to be as inclusive as possible in recruitment so that the intervention we are testing is applicable to most people living with Parkinson’s. The intervention was designed as being largely digital, facilitated by remote sessions with a supporter, which reduces the cost of the intervention and allows for easier implementation across geographically dispersed healthcare settings. Certain groups, however, experience greater difficulty engaging with self-management interventions and accessing digital healthcare [48, 49]. We therefore have developed a paper-based manual alternative (which can also be used alongside the digital version if preferred), that can be facilitated by face-to-face or telephone sessions with a supporter with an interpreter if needed and involving an informal carer/friend or family member. In our process evaluation, we will explore the experiences of people receiving this different tailored intervention compared to the digital Toolkit and approaches to maximise engagement for people who find self-management and digital healthcare interventions more difficult.

## Limitations

As the ‘Live well with Parkinson’s’ Toolkit is a self-management intervention, participants cannot be blinded to arm allocation, which may bias participant-reported outcomes. Half of our outcome assessments are interviewer-administered and researchers carrying out assessments and key trial management staff will be blinded to arm allocation to reduce the risk of bias. Additionally, more ‘supporters’ will be delivering the intervention sessions than in the feasibility study, which may affect consistency across participants or sites. To reduce the risk of drift, the supporters will have regular case-based supervision and fidelity of delivery will be explored in the process evaluation.

## Conclusion

This RCT represents an important step forward in the development and evaluation of self-management interventions for people with Parkinson’s. By bringing together the expertise of people with lived experience, health and social care practitioners, and researchers, this intervention has been designed to meet the needs of diverse populations and has the potential to improve the quality of life for people living with Parkinson’s.

### Supplementary Information

**Additional file 1.**

**Additional file 2.**

**Additional file 3.**

**Additional file 4.**

## Data Availability

The datasets generated during and/or analysed during the current study are/will be available upon request from PRIMENT CTU Data Management Group and will only be possible in collaboration with members of the PD-Care Trial Management Group. Please contact Anne Marie Downey, the Head of Clininical Trials Operations at PRIMENT CTU, at a.downey@ucl.ac.uk to request data from this study. The data underlying this study contains sensitive information and cannot be made publicly available, according to PRIMENT CTU Data Management Group. PRIMENT CTU is a UK Clinical Research Collaboration (UKCRC) registered clinical trials unit and is based at UCL (see here for more information: https://www.ucl.ac.uk/priment). The study collects quantitative data from patient assessments as well as interview data, which will become available after data processing and cleaning. It will be made available to bona fide researchers upon reasonable request, observing data protection rules.
