# Supplementary material for: Clinical and cost-effectiveness of ‘Live Well with Parkinson’s’ self-management intervention versus treatment as usual for improving quality of life for people with Parkinson’s: study protocol for a randomised controlled trial
Source: Trials. 2023 Dec 5;24:793. doi: 10.1186/s13063-023-07700-7 (PMC10698895; doi:10.1186/s13063-023-07700-7)
Supplement: Supplementary file 2 — Additional file 2. [file 13063_2023_7700_MOESM2_ESM.docx]

**Supplementary file 1. TIDIER checklist for Live Well with Parkinson’s**

| **TIDIER item** | **Application in Live Well with Parkinson’s** |
| --- | --- |
| 1 Brief name: Provide the name or a phrase that describes the intervention | Live Well with Parkinson’s |
| 2 Why: Describe any rationale, theory, or goal of the elements essential to the intervention | An asset-based approach underpins the overall intervention approach, focussing on maintaining independence, health and current activities rather than addressing deficits. Use of the self-management toolkit is to empower individuals and for them to have more control over their healthcare and condition, by learning new skills to manage their Parkinson’s. We used Corbin and Strauss’s model of self-managing a long-term condition. In this model there are three self-management tasks to address: medical, role, and emotional management. A further six self-management skills have been described: problem solving, decision making, resource utilisation, formation of a patient-provider partnership, action planning and self-tailoring. The COM-B model (Capability, Opportunity Motivation – Behaviour) is the foundation of the goal setting process to identify each participant’s barriers to behaviour change and build strategies into the action plan to overcome these barriers. |
| 3 What materials: Describe any physical or informational materials used in the intervention, including those provided to participants or used in intervention delivery or in training of intervention providers. Provide information on where the materials can be accessed (such as online appendix, URL) | - Toolkit (available on paper and online) consisting of 64 sections of information to read, self-management guidance and resources and several personalised sections including a symptom review, goal setting and action planning, monitoring (symptom/medication tracker), calendar function with e-mail reminder-system. - Live Well with Parkinson’s intervention manual for provider training to deliver supporter sessions. - Training materials include a combination of self-directed pre-recorded lectures and activities and structured live sessions, on the topics of Parkinson’s, goal setting, behaviour change, and psychological wellbeing. - Supporter checklists to be completed following each intervention session, which can be found in the intervention manual. - Phone or video calling is required for remote delivery. Internet-enabled tablets may also be provided to participants with no access to video calling. |
| 4 Procedures: Describe each of the procedures, activities, and/or processes used in the intervention, including any enabling or support activities | 1. Access to the Toolkit (web-based or paper versions or both if preferred), with a personal log-in to securely store personal information. Separate log-ins with ability for the person with Parkinson’s to share information with their carer and designated health care professional also available. 2. Up to six supporter sessions to facilitate the intervention, which includes the following:  - First appointment (60-90 min): build rapport and learn about the participant; introduce the toolkit and various sections, and the intervention sessions; identify their aim from using the toolkit and create a SMART goal. - Second appointment (30-60min): this session reintroduces all sections to facilitate understanding with a main focus on the ‘My Wellbeing’ section. If a SMART goal was not set at the end of the first appointment, this appointment includes SMART goal setting. If a goal was set, this appointment focuses on reviewing progress towards goals, providing feedback, forming habits, modifying goals as needed to overcome barriers or selecting new goals. If needed, the supporter will explore ways to cope with setbacks or build motivation and any troubleshooting needed for the toolkit. - Subsequent appointments (3-5, 30-45min): Reviewing progress towards goals with modifications or signposting as needed, as described above. - Final appointment (6, 30min): reinforce self-efficacy, remind the participant of new things they have learned and achieved, advice and planning for maintaining motivation and forming habits, information on further help and support. GP summary sheet is completed and sent to the GP to update on progress in the study. Access to the Toolkit continues after the final appointment is completed.   Contact may be made between appointments to provide information or confirm appointments, but this is expected to be limited.  Behaviour change functions include education, training, enablement and environmental restructuring (e.g. helping the person to access home modifications or assistive equipment to enable them to continue activities or reach a goal). Supporters deliver a core set of self-regulatory behaviour change techniques (BCTs) to each participant: outcome goal setting (if appropriate), behavioural goal setting, action planning, problem solving, reviewing progress (i.e. review outcome goal [if appropriate]; discrepancy between current behaviour and goal; review behavioural goal), and provide feedback on behaviour. Additional BCTs drawn from the ‘Goals and Planning’ and ‘Feedback and Monitoring’ BCT Taxonomy v1 clusters (e.g., encouraging self-monitoring) are used where applicable. |
| 5 Who provided: For each category of intervention provider (such as psychologist, nursing assistant), describe their expertise, background, and any specific training given | Supporters: non-specialist workers with experience in working with people with Parkinson’s, who are specifically trained for this role. Typically, they may have a background working in the voluntary sector, social prescribing, or nursing, but are not required to have a particular qualification.  The supporters will receive six days of training including two days learning about Parkinson’s, two days learning about the intervention manual and the toolkit, and two days learning behaviour change skills such as motivational interviewing and goal development. The training will be conducted by a mixture of behaviour change experts and neurologists, as well as provided with access to the information available from the toolkit and from Parkinson’s UK website. Supporters receive supervision, every 2-4 weeks, each lasting around 30 minutes from a supervisor with expertise in behaviour change. |
| 6 How: Describe the modes of delivery (such as face to face or by some other mechanism, such as internet or telephone) of the intervention and whether or not it was provided individually or in a group | The Toolkit can be accessed via a website that has been optimised for smart phone and tablet access, or as a paper-based manual, which is posted to participants.  Supporter sessions are typically video calling or telephone appointments. Face-to-face individual appointments can be given if the participant requests this. A carer may also be present where the person wishes or if they have significant cognitive impairment. |
| 7 Where: Describe the type(s) of location(s) where the intervention occurred, including any necessary infrastructure or relevant features | The intervention is largely remote, with a digital or paper-manual Toolkit and video-calling or telephone supporter sessions. In instances where face-to-face appointments are requested these can be in clinic settings if available/preferred or participants’ homes, requiring time and budget for travel and secure storage of notes and health and wellbeing plans during transit. If delivered remotely, participants require a phone or internet-enabled device. |
| 8 When and how much: Describe the number of times the intervention was delivered and over what period of time including the number of sessions, their schedule, and their duration, intensity or dose | Access to the Toolkit (digital via website login or a manual) for 12 months.  The Toolkit is facilitated with supporter sessions of up to six sessions over a six-month period, with some flexibility as to the timing and amount. Three appointments are considered a minimum dosage. The first appointment is approximately 1-2 hours and subsequent appointments last around 30 min (max 60min). |
| 9 Tailoring: If the intervention was planned to be personalised, titrated or adapted, describe what, why, when, and how | The intervention is tailored according to the needs and goals of the participant. Behaviour change techniques selected (see Section 4 above) are also tailored, with COM-B used to identify individual potential barriers to overcome and these are built into the ‘My Wellbeing’ section of the toolkit. The total number and duration of appointments follows the guide outlined above but can be tailored within this guide as needed. It is anticipated that not all participants will need all six supporter sessions. |
| 10 Changes: If the intervention was modified during the course of the study, describe the changes (what, why, when, and how) | n/a |
| 11 How well – planned: If intervention adherence or fidelity was assessed, describe how and by whom, and if any strategies were used to maintain or improve fidelity, describe them | Fidelity: self-reported supporter checklists, plus independent rating of audio recorded appointments with 10% of those receiving the intervention. No feedback is given to supporters during the intervention regarding the audio-recording fidelity rating. Intervention supporters receive regular supervision as described above to maintain fidelity. |
| 12 How well – actual: If intervention adherence or fidelity was assessed, describe the extent to which the intervention was delivered as planned | n/a  This will be evaluated in our planned parallel process evaluation. |
