# Supplementary material for: Clinical and cost-effectiveness of ‘Live Well with Parkinson’s’ self-management intervention versus treatment as usual for improving quality of life for people with Parkinson’s: study protocol for a randomised controlled trial
Source: Trials. 2023 Dec 5;24:793. doi: 10.1186/s13063-023-07700-7 (PMC10698895; doi:10.1186/s13063-023-07700-7)
Supplement: Supplementary file 3 — Additional file 3. [file 13063_2023_7700_MOESM3_ESM.docx]

PERSONALISED CARE FOR PEOPLE WITH PARKINSON’S DISEASE: PD-CARE

Statistical Analysis Plan

Version 1.0

22 September 2023

# Introduction

## Purpose and scope of the statistical analysis plan

This statistical analysis plan (SAP) contains details of the main pre-specified statistical analyses for the PD-CARE trial. This SAP describes the statistical analysis of the clinical outcomes. The SAP does not preclude the undertaking of further ad hoc or exploratory analyses, although the results of any such analyses should be interpreted with caution. Furthermore, the SAP does not preclude the adaptation of any part of the trial analysis should situations arise in which such adaptation is deemed necessary; any such adaptation will be transparent and fully justified. This SAP does not contain details of analyses related to health economics and process evaluation; these are described in separate documents.

## Protocol version

Full details of the trial design, population, intervention, comparison and outcome variables may be found in the protocol (version 1.4 dated 23/03/2023).

## Trial registration

The trial is registered with the ISRCTN registry (<https://doi.org/10.1186/ISRCTN92831552>).

## Authorship

This SAP has been written by Mariam Adeleke (MA) and Gareth Ambler (GA), following the guidelines of Gamble *et al* (2017).

## SAP Revisions

| Version | Date | Changes |
| --- | --- | --- |
| 1.0 | 22.09.23 |  |

# Trial Summary

## Title

Personalised care for people with Parkinson’s Disease: PD-Care

## Aims

The study objectives are to evaluate the effectiveness and cost-effectiveness of the “Live Well with Parkinson’s” facilitated self-management toolkit, which aims to enable personalised care for community-living people with Parkinson’s, to reduce disability and preventable hospital admissions and to improve quality of life.

The specific objectives are to:

1. Determine the clinical effectiveness of the ‘Live Well with Parkinson’s’ intervention (facilitated self-management toolkit) through a definitive Randomised Controlled Trial, with internal pilot.
2. Determine the cost-effectiveness of the intervention from the perspective of the NHS and personal social services.
3. Determine the factors promoting or inhibiting implementation of the toolkit in the NHS.

## Population

Community-dwelling people with Parkinson’s disease.

### Inclusion criteria

Community-dwelling adults with a confirmed diagnosis of Parkinson’s Disease (PD), (defined using UK Brain Bank Criteria (89)), including those with dementia diagnosed at least one year after PD.

### Exclusion criteria

- Atypical Parkinsonism
- Currently an inpatient or living in a care home
- Lack of capacity to take part MoCA <11
- Life expectancy <6months

# Study Methods

## Design

A multi-centre, single blind trial, parallel group, two arm, randomised controlled trial to assess the clinical and cost effectiveness of PD-CARE in people with Parkinson’s disease with a 6-month internal pilot.

## Intervention

The usual care plus training and access to PD-Care, supported by trained service providers (nurses independent of their usual care to prevent contamination of the control arm) for up to 6 sessions.

## Comparison

The usual care from existing sources (GP, PD specialist service +/- NHS PD Nurse Specialist).

## Sample size

To detect a 4.7-point difference in PDQ-39 with 90% power and 5% significance, 135 participants per arm are required, assuming a SD of 19.8 and a correlation between baseline and follow-up measurements of 0.8. Allowing for 20% attrition at 12 months increases the total to 338 people with Parkinson’s.

## Randomisation

Randomisation of study participants will be performed by PRIMENT CTU using a web-based service Sealed Envelope in a 1:1 ratio to the intervention or treatment as usual (TAU). Minimisation will be used to perform individual randomisation based on site and disease severity (early vs advanced disease).

## Blinding

This is a single blind trial. Assessors are blind to treatment allocation; participants are not. Statisticians and health economists will also be blinded to allocation as far as possible until after the primary analysis has been agreed. One of the statisticians will attend the Data Monitoring and Ethics Committee part of the Trial Steering Committee (TSC) and consequently may become unblinded if the committee requires any statistics to be reported separately by study arm.

# Statistical Principles

## Organisation of data and analyses

The SAP will be finalised and approved prior to unblinding. The programs and code to be used for statistical analyses will be prepared prior to unblinding as far as possible. Two statisticians will perform the analysis relating to the primary outcome independently, in order to ensure its accuracy.

Prior to performing analyses, basic checks will be performed by the statisticians on the blinded data to ensure accuracy. Each outcome (primary and secondary) variable and baseline demographic variable will be checked for:

- missing values
- values outside an acceptable range
- other inconsistencies

If missing values or other inconsistencies are found, the corresponding data will be sent to the Trial Manager for checking and will either be corrected, deemed to be missing or confirmed correct, as appropriate.

## Confidence intervals and p-values

All statistical tests will be two-sided. All estimates will be presented with two-sided 95% confidence intervals.

## Adherence to intervention supporter sessions

For the main analyses, adherence is defined as attending at least 3 intervention supporter sessions. Other definitions of adherence may be used for exploratory analyses.

## Analysis populations

The ‘intention-to-treat’ population will include all randomised patients according to the treatment to which they were randomised to receive. Any patients that have withdrawn from the trial, and withdrawn permission to keep and use their data, will be necessarily excluded.

# Trial Population

## Recruitment and retention

A CONSORT diagram will be presented to provide a detailed description of participant numbers at each time point during the trial. In addition, a table summarising the number of participants who have been lost to follow up at each stage of the trial and reasons for loss to follow up (if supplied) will be presented.

## Baseline Characteristics

The demographic information and other questionnaire scores collected at baseline will be presented in a table summarised separately by study arm. Categorical variables will be reported as counts and percentages. Continuous variables will be summarised as means and standard deviations (SD) or medians and interquartile ranges as appropriate depending on the distribution of the data. No statistical tests will be performed to assess baseline differences between study arms. In addition, all baseline outcomes (see Table 1) will be presented in a table summarised separately by study arm.

The following characteristics will be summarised:

- age
- gender
- ethnicity
- marital status
- living arrangements
- employment status and time since last paid employment
- disease duration and severity
- baseline outcomes

Other available data will comprise:

- centre identifier
- dates of assessments

# Statistical analysis plan

## Outcomes

Below we list the outcomes that will be analysed as part of the main statistical analysis. Outcomes that will be used for the Health Economics and Process Evaluation analyses are not listed, though they appear in Table 1. In addition, the motion sensor and saliva outcomes are not listed as they will be analysed later, as part of a separate statistical analysis.

### Primary outcome

The primary outcome is Parkinson’s Disease Questionnaire (PDQ-39) score at 12 months.

### Secondary outcomes

All secondary outcomes will be analysed at all available time-points (typically 6 and 12 months, though see Table 1 in Section 6.1.4). These outcomes are:

- Parkinson’s Disease Questionnaire (PDQ-39) score at 6 months
- Movement Disorders Society - Unified Parkinson’s Disease Rating Scale (MDS-UPDRS) Part I and II
- Off-time, MDS-UPDRS part 3 (motor), part 4 (motor-complications) and total score
- Non-Motor Rating Scale (MDS-NMS)
- Psychological well-being (GHQ12, 12 items)
- Zarit carer burden inventory
- Carer quality of life questionnaire for Parkinsonism

### Safety outcomes

- Serious Adverse Events / Adverse Events
- Deaths

### Timing of outcomes

Table 1 provides an overview of primary and secondary outcomes and the time points at which they will be collected. This analysis will only describe the analysis of those outcomes listed under ‘Statistical Outcomes’.

Table 1: Data collection measures and time points (from Table 1 in the Protocol).

|  | Baseline | 6 months | 12 months |
| --- | --- | --- | --- |
| People with PD | | | |
| *Statistical Outcomes* |  |  |  |
| PDQ-39 | X | X | X |
| Montreal Cognitive Assessment test (MoCA) | X |  | X |
| Unified Parkinson’s Disease Rating Scale (MDS-UPDRS) | X | X | X |
| Non-Motor Rating Scale (MDS-NMS) | X | X | X |
| General Health Questionnaire (GHQ-12) | X | X | X |
| *Health Economics Outcomes* |  |  |  |
| EQ-5D-5L | X | X | X |
| Client Service Receipt Inventory - adapted for PD (CSRI) | X | X | X |
| ICECAP-O | X | X | X |
| *Process Evaluation Outcomes* |  |  |  |
| Self-Efficacy for Managing Chronic Disease | X | X | X |
| Patient Activation Measure (PAM) | X | X | X |
| *Other Outcomes* |  |  |  |
| Saliva sample (one time point only) | X | X | X |
| Motion sensor | X | X | X |
| Carers | | | |
| *Statistical / Process Evaluation Outcomes* |  |  |  |
| Zarit carer burden inventory | X | X | X |
| Carer Quality of Life questionnaire for Parkinsonism | X | X | X |

## Primary outcome analysis

The primary outcome is PDQ-39 score at 12 months. The primary analysis will compare intervention patients to control patients using a three-level mixed model that includes the PDQ-39 scores from two time-points (6 months and 12 months) with an interaction term between the intervention and time-point indicator variables to enable estimation of the intervention effect at 12 months. This model will also adjust for baseline PDQ-39 score, age and socio-economic status using fixed effects, and will adjust for patient and site using random effects (random intercepts). All analyses will be performed on an intention-to-treat basis and all modelling assumptions will be checked (e.g. using residuals). The effect of intervention and corresponding 95% confidence interval will be reported.

## Secondary outcome analysis

The effect of the intervention on secondary outcomes will be assessed using appropriate two-level regression models, i.e. linear mixed models for numerical outcomes. All models will be adjusted for age, socio-economic status and baseline outcome (if available) using fixed effects, and site using random effects (random intercepts). The effect of intervention and corresponding 95% confidence interval will be reported., though P-values will not be reported for secondary analyses. These analyses will be considered supportive.

## Sensitivity analyses

Several sensitivity analyses will be performed for the primary outcome. These are:

- An analysis may be performed to adjust for any baseline imbalance caused either by chance or by missing data (see Section 6.6).
- A complier average causal effect (CACE) analysis and per-protocol analysis will be performed to adjust for any non-adherence to the intervention (see also Section 6.7). Adherence to the intervention supported-sessions is defined as attendance at 3 intervention sessions (from Section 4.3).

## Subgroup analysis

We will undertake a pre-specified subgroup analysis exploring the effectiveness in early (diagnostic/maintenance) vs. advanced (complications/palliative) PD. Three groups will be defined for this analysis based on Hoehn and Yahr stages (1-2 vs 3 vs 4-5), though groups may be combined if numbers are small (i.e. 3 may be combined with 4-5). This analysis will be performed by adding a main effect for subgroup and interaction terms between subgroup and intervention to the primary analysis model.

## Missing data

Withdrawals from the study, loss to follow up and other missing outcome data will be summarised separately by randomised group. Potential bias due to missing data will be investigated by comparing the baseline characteristics of participants with and without missing values. Depending on the quantity of missing values, predictors of missingness may be identified. We will then perform a sensitivity analysis that includes these predictors of missingness as covariates in the primary analysis model.

Multiple imputation may also be performed, if deemed appropriate. The imputation model will include outcome data from all time-points, as well as baseline characteristics. The primary analysis model will then be re-fitted using the imputed data.

In addition, imputation may be performed under the assumption that the missing data are MNAR. Two strategies may be investigated:

- Delta-adjustment: This approach initially uses (standard) multiple imputation to impute missing values but then the imputed values are modified using a ‘delta-adjustment’. Different values of delta may be specified, and these can differ by trial arm.
- Reference-based sensitivity analyses: A range of different assumptions regarding the missing data can be investigated using this approach, which is implemented in the Stata package **mimix** (Cro *et al*, 2016).

## Additional analyses

Several additional analyses may be performed. These are:

- The effect of the intervention at 12 months will be assessed using a two-level linear mixed model, adjusted for age, socio-economic status and baseline PDQ-39 score using fixed effects, and site using random effects (random intercepts).
- Adherence to intervention will be described, e.g. in terms of the mean (SD) number of sessions attended. Reasons for withdrawal or loss to follow-up will be described.

## Adverse events

The number, nature and severity of serious adverse events (if any) will be reported separately by study arm at each follow up time point. The number of participants who experience adverse events will likewise be reported separately by study arm.

## Reporting

Analyses will be reported with regard to the CONSORT checklist and with any particular requirements of academic journals and the funders to which the results of analyses are submitted.

# Appendix

## List of Abbreviations

| AE | Adverse Event | MOCA | Montreal Cognitive Assessment test |
| --- | --- | --- | --- |
| AR | Adverse Reaction | NICE | The National Institute for Health and Care Excellence |
| CACE | Complier Average Cause Effect | NMSS | Non-Motor Rating Scale |
| CI | Chief Investigator | PD | Parkinson’s disease |
| CRF | Case Report Form | PI | Principle Investigator |
| CRO | Contract Research Organisation | PD-QOL | Parkinson’s disease quality of life |
| CSRI | Client Service Receipt Inventory-shortened, adapted for PD | PIS | Participant Information Sheet |
| DMC | Data Monitoring Committee | QA | Quality Assurance |
| EQ-5D-5L | Quality of life measure | QALY | Incremental cost per quality adjusted life year |
| GCP | Good Clinical Practice | QC | Quality Control |
| GDPR | General Data Protection Regulation | RCT | Randomised Controlled Study |
| GAfREC | Governance Arrangement for NHS Research Ethics | REC | Research Ethics committee |
| IB | Investigator Brochure | SAR | Serious Adverse Reaction |
| HCP | Healthcare professional | SAE | Serious Adverse Event |
| ICECAP-O | Capability measure | SDV | Source Data Verification |
| ICF | Informed Consent Form | SOP | Standard Operating Procedure |
| ISRCTN | International Standard Randomised Controlled Studies Number | SSI | Site Specific Information |
| GHQ12 | General Health questionnaire: Short form | TAU | Treatment as usual |
| MDS-UPDRS | Movement Disorders Society-Unified Parkinson’s Disease Rating Scale | UCL | University College London |

# References

- Gamble et al. Guidelines for the Content of Statistical Analysis Plans in Clinical Trials. JAMA 2017; 318(23):2337-2343.
- Cro S, et al. Reference-based sensitivity analysis via multiple imputation for longitudinal trials with protocol deviation. Stata Journal 2016; 16(2):443-63.
