# Supplementary material for: Clinical and cost-effectiveness of ‘Live Well with Parkinson’s’ self-management intervention versus treatment as usual for improving quality of life for people with Parkinson’s: study protocol for a randomised controlled trial
Source: Trials. 2023 Dec 5;24:793. doi: 10.1186/s13063-023-07700-7 (PMC10698895; doi:10.1186/s13063-023-07700-7)
Supplement: Supplementary file 4 — Additional file 4. [file 13063_2023_7700_MOESM4_ESM.zip › Anonymised PIS Carer Supp 2R1.docx]

**Carer Information Sheet**

**(Randomised Controlled Trial)**

**Name of Lead Researchers:** [Add name here]

**Name of Site Principal investigator**: [Add name here]

We would like to invite you to take part in our research study. Before you decide whether you want to take part, it is important that you understand why the study is being done and what participating in the study entails. One of our team will go through this information sheet with you, to help you decide whether you would like to take part or not and answer any questions you may have. We'd suggest this should take about 45 minutes. Please read the following information carefully and please feel free to talk to others about the study if you wish.

Part 1 tells you the purpose of this study and what will happen to you if you take part.

Part 2 gives you more detailed information about the conduct of the study.

Part 3 gives you more in-depth information on your data rights and how we use your data.

Please ask us if there is anything that is not clear and do take time to decide whether you wish to take part.

**Part 1 Purpose of this study and what will happen to you if you take part**

1. **What is the purpose of the study?**

This study aims to improve the care of people with Parkinson's by testing the effectiveness of the Live Well with Parkinson’s Toolkit compared to treatment as usual. The toolkit is available online and in paper and aims to help increase the active involvement of people with Parkinson’s in the management of their care, including how to keep healthy and independent, where to access resources and how to manage their Parkinson’s. The toolkit is facilitated by a ‘Supporter’ who will offer up to six one-to-one sessions with the participants in which they will be directed to relevant information and advice on symptoms that may be bothering them as well as identifying what living well means to them and what steps they can take to achieve this. It is hoped that the *Live Well with Parkinson’s* toolkit will reduce disability and hospital admissions and improve day-to-day living and quality of life. If successful, this toolkit could then be used across the NHS to be available to help as many people as is possible.

1. **Why have I been invited?**

You have been invited because you are the partner or carer of someone who has a diagnosis, or possible diagnosis, of Parkinson’s and they have nominated you to take part in this study. We are inviting up to 338 people to take part in this stage of the study.

1. **Do I have to take part?**

No, participating in this study is voluntary. If you prefer not to take part in the study, this will not influence the current care your partner or carer recipient currently receives from doctors, nurses, or any other healthcare professionals. If you agree to take part, we will then ask you to sign a consent form. If you agree to take part but then change your mind you can withdraw from the study at any time without giving a reason.

1. **What will I have to do if I take part?**

- Before taking part in the study a member of the research team will discuss the study in detail with you and give you time to ask any more questions that you may have. If eligible to take part in the study, you will then be asked to sign a consent form to confirm that you are happy to take part.
- Taking part in the study will involve completing questionnaires that take around 15 minutes on three separate occasions throughout a year; at the start, after 6 months and after 12 months. The questionnaires will ask about being a carer or supporter of someone with Parkinson’s.

1. **Expenses and payments**

If you agree to take part in the study you will be offered a £20.00 high street shopping voucher as a thank you for your time for the first assessment, and £10 for each of the two further shorter assessments that you complete (£40 in total for completing all three assessments). We will also reimburse any travel expenses you may have.

1. **What are the possible disadvantages and risks of taking part?**

We do not envisage any disadvantages in taking part in the study, but it will require a commitment of your time.

1. **What are the possible benefits of taking part?**

Whilst there will be no direct benefit of taking part in this stage of the study, it is hoped that the toolkit and training developed from this research will be of help to those living with Parkinson’s. If found to be effective, it is hoped that the *Live Well with Parkinson’s Toolkit* will improve quality of life, reduce disability and unnecessary hospital admissions; and help health care professionals in the delivery of the best care.

1. **What happens when the research study stops?**

The study will not affect your partner or care recipient’s usual Parkinson’s treatment and therefore, when the study is completed they will continue their usual treatment.

This completes Part 1 of the Information Sheet.

If the information in Part 1 has interested you and you are considering participation, please read the additional information in Part 2 before making any decision.

**Part 2: Information about the conduct of the study**

1. **What will happen if I don’t want to carry on with the study?**

You can withdraw from the study at any time without giving a reason.

1. **What if there is a problem?**

Every care will be taken during this study to ensure that your well-being is not compromised. If however you or your relatives have any concerns about any aspect of the way you have been approached or treated by members of staff during this study you can speak to a member of the research team who will do their best to answer any questions. Their contact details are at the end of this information sheet.

If you remain unhappy and wish to complain formally, you can do this by contacting the confidential patient advice and liaison service (PALS). PALS was set up to support patients, their families and visitors who need advice or have problems and concerns. [Add PALS details here]

In the unlikely event that something does go wrong, and you are harmed during the research due to someone's negligence then you may have grounds for a legal action for compensation against [Hospital Site], but you may have to pay your legal costs. The normal National Health Service complaints mechanisms will still be available to you.

1. **Will my taking part in this study be kept confidential?**

[Add sponsor name] is the sponsor of this study and is based in the United Kingdom. We will be using information from you/ and your medical records to undertake this study and will act as the data controller for this study. This means that we are responsible for looking after your information and using it properly. [Add sponsor name] will keep identifiable information about you for 5 years after the study has finished.

Your rights to access, change or move your information are limited, as we need to manage your information in specific ways for the research to be reliable and accurate. If you withdraw from the study, we will keep the information about you that we have already obtained. To safeguard your rights, we will use the minimum personally-identifiable information possible.

You can find out more about how we use your information by contacting study members listed at the end of this information sheet and/or [Add details].

1. **What will happen to the results of the research study?**

The scientific results of the study will be available after it finishes and will usually be published in a medical journal or be presented at a scientific conference. The data will be anonymous and none of the participants involved in the study will be identified in any report or publication. Should you wish to see the results, or the publication, please sign the relevant section of the consent form.

1. **Who is organising and funding the research?**

[Add sponsor name] is the sponsor for the study and the study is funded from a grant from the National Institute of Health Research (NIHR). None of the doctors or researchers who are working on this study are being paid for including participants in the study, and there are no conflicts of interest.

1. **Who has reviewed the study?**

All research in the NHS is looked at by an independent group of people, called a Research Ethics Committee, to protect your interests. This study has been reviewed and given favourable opinion by [Add ethics committee name].

1. **Further Information and contact details**

You are encouraged to ask any questions you wish, before, during or after the study. If you have any questions about the study, please speak to a member of the research team who will be able to provide you with up to date information. If you require any further information or have any concerns while taking part in the study please contact one of the following people:

**Trial Manager**: [Add name and email]

**Research Assistants**: [Add name(s) and email(s)]

**Study email address:** [Add email]

Alternatively if you or your relatives have any questions about research or Parkinson’s you may wish to contact one of the following organisations that are independent of the hospital at which you are being seen: Parkinson’s UK’s website [www.parkinsons.org.uk](http://www.parkinsons.org.uk), the NIHR website [www.peopleinresearch.org](http://www.peopleinresearch.org) or [www.invo.org.uk](http://www.invo.org.uk)

**Part 3: In-depth information on your data rights and how we use your data**

**Your data rights and how we use your data**

Your data belongs to you, so it's important that it’s only used in ways you’d reasonably expect, and that it stays safe. Data protection law makes sure everyone’s data is used properly and legally.

**How does participating involve my personal data?**

- Relevant sections of medical notes and data collected during the study may be looked at by individuals from the [Add details] or the research team at UCL. Information collected about you will be used to support other research in the future and may be shared anonymously with other researchers.
- The Unified Parkinson's Disease Rating Scale (UPDRS) and supporter sessions are video, or audio recorded to ensure accurate assessment and correct delivery. These recordings may be transcribed (written down). The recordings are deleted from the recording device and kept securely. No images or audio of you will appear anywhere without your permission.
- Optional saliva samples will be stored until used in future research studies. Samples will be stored and treated in line with the rules of the [Human Tissue Authority](https://www.hta.gov.uk/guidance-public/body-organ-and-tissue-donation/donating-your-tissue-research), to be analysed and used in future studies that are approved by an Ethics Committee.

**Your Rights**

| 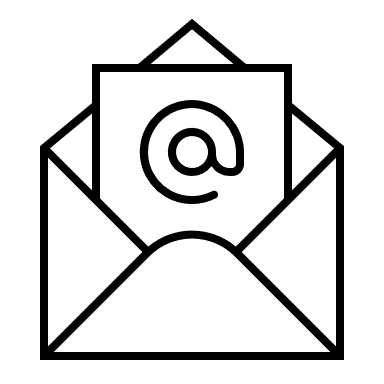[Get copies of your data](https://ico.org.uk/your-data-matters/your-right-to-get-copies-of-your-data/) | 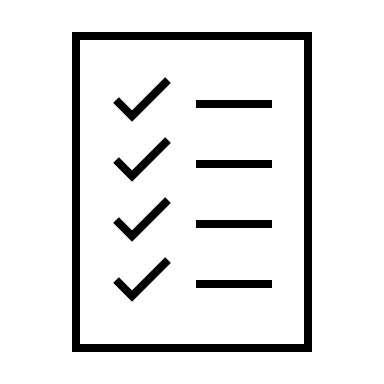[Get your data corrected](https://ico.org.uk/your-data-matters/your-right-to-get-your-data-corrected/) |
| --- | --- |
| 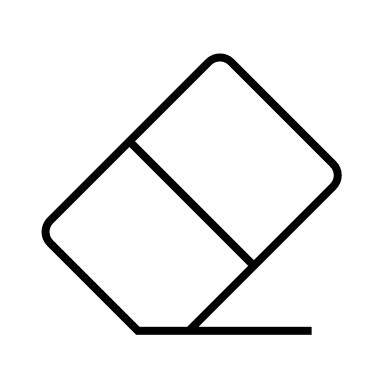[Get your data deleted](https://ico.org.uk/your-data-matters/your-right-to-get-your-data-deleted/) | 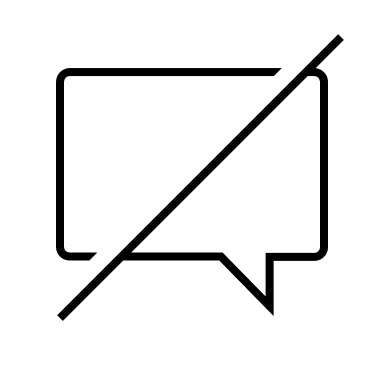[Limit how we use your data](https://ico.org.uk/your-data-matters/your-right-to-limit-how-organisations-use-your-data/) |
| 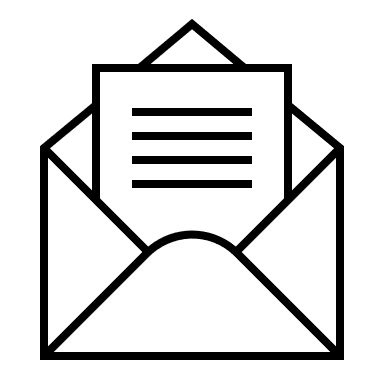[Get your data in an accessible way](https://ico.org.uk/your-data-matters/your-right-to-data-portability/) | 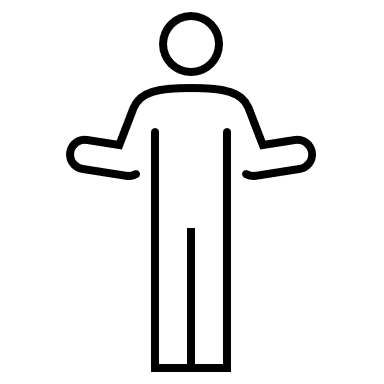[Object to the use of your data](https://ico.org.uk/your-data-matters/the-right-to-object-to-the-use-of-your-data/) |
| 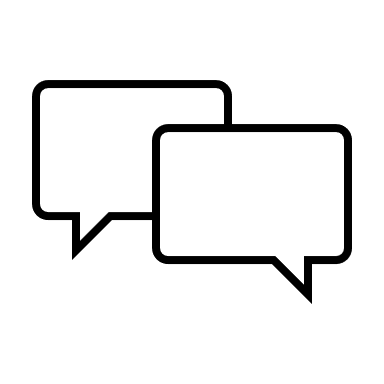[Make data protection complaint(s)](https://ico.org.uk/your-data-matters/how-to-make-a-data-protection-complaint/) | 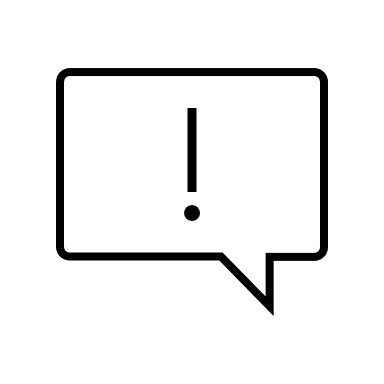[Be informed if personal data is being used](https://ico.org.uk/your-data-matters/your-right-to-be-informed-if-your-personal-data-is-being-used/) |
| 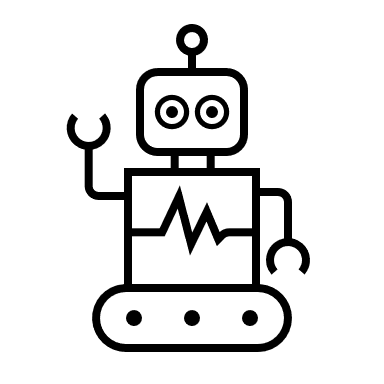[Object to automated processing](https://ico.org.uk/your-data-matters/your-rights-relating-to-decisions-being-made-about-you-without-human-involvement/) | 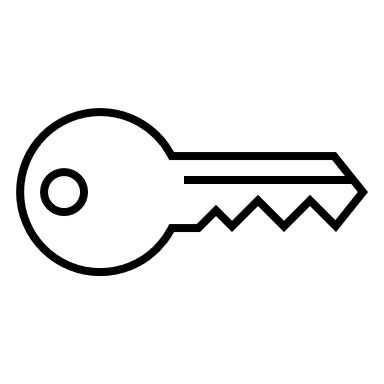[Access information from a public body](https://ico.org.uk/your-data-matters/official-information/) |

**Ways we secure your data**

1. **Authorised Users:** Only approved staff working on the study can access information.
2. **Anonymise data:** We anonymise as much data as possible so people cannot be identified.
3. **Encrypted USBs:** Any data that's moved to an external device is encrypted, meaning that only authorised staff members can read the information stored.
4. **Data Safe Haven:** Any information that isn't anonymised is stored on secure servers that restrict who can access it, uses multiple passwords for login, and prevents any information from being downloaded or moved.

**Why do we use patient data?** In clinical trials, researchers are collecting data that will tell them whether one treatment is better or worse than the other. The information collected will show how safe a treatment is, or whether it is making a difference to your health. Different people can respond differently to a treatment. By collecting information from lots of people, researchers can use statistics to work out what effect treatment is having.

**How do we use patient data?** If you take part in some types of research, like clinical trials, some of the research team will need to know your name and contact details so they can contact you about your research appointments, or send you questionnaires. Researchers must always make sure that as few people as possible can see this sort of information that can show who you are.

**What happens to my data after the study?** Researchers must make sure they write the reports about the study in a way that no one can work out who took part in the study. Once they have finished the study, the research team will keep the research data for several years, in case they need to check it. You can ask about who will keep it, whether it includes your name, and how long they will keep it for.

**What if I don’t want my data used for research?** You will have a choice about taking part in a clinical trial testing a treatment. If you choose not to take part, that’s okay! In most cases, you will also have a choice about your patient data being used for other types of research. There are two cases where this might not happen:

1. When the research is using anonymous information. Because it's anonymous, the research team doesn't know whose data it is and can't ask you.
2. When it would not be possible for the research team to ask everyone. This would usually be because of the number of people who would have to be contacted. Sometimes it will be because the research could be biased if some people chose not to agree. In this case, a special NHS group will check that the reasons are valid. You can opt out of your data being used for this sort of research.

**Freedom of Information**

The Freedom of Information (FOI) Act 2000 imposes a duty on public authorities: (1) To confirm or deny that the information requested is held. (2) If the information is held, communicate it to the applicant. Anyone may make a request in writing for recorded information held by or on behalf of any organisation, and they must comply promptly and in any case within 20 working days.  Unless subject to one of 23 exemptions described by the Act, the information must be provided.

- [Add contact details]

Enquiries about use of data can be directed to [Add email] or contact [Add details] to speak with a session supporter.

UCL’s data protection officer can be contacted through [Add details].  Report a data breach or make a complaint by visiting the information commissioner's office website: [Add web address] or calling their helpline on [Add number].

Thank you for taking the time to read this information sheet and for considering taking part in this study. You can have more time to think this over if you are at all unsure. If you decide you would like to take part, then please read and sign the consent form provided by the research team. You will be given a copy of this information sheet and the signed consent form to keep. A copy of the consent form will be filed in your patient notes, and one will be filed with the study site files.

| **Chief Investigators [Add name and contact details]** |  |
| --- | --- |
