# Supplementary material for: Clinical and cost-effectiveness of ‘Live Well with Parkinson’s’ self-management intervention versus treatment as usual for improving quality of life for people with Parkinson’s: study protocol for a randomised controlled trial
Source: Trials. 2023 Dec 5;24:793. doi: 10.1186/s13063-023-07700-7 (PMC10698895; doi:10.1186/s13063-023-07700-7)
Supplement: Supplementary file 4 — Additional file 4. [file 13063_2023_7700_MOESM4_ESM.zip › Anonymised Carer Consent Form_supp 2R1.doc]

Study Number:

**Carer Consent Form**

**(Randomised Controlled Trial)**

| **Name of Lead Researchers**: [Add name here]  **Name of Site Principal investigator**: [Add name here] | | Please initial each question | |
| --- | --- | --- | --- |
| 1 | I confirm that I have read and understood the information sheet dated.................... (version............) for the above study. I have had the opportunity to consider the information, ask questions and have had these answered satisfactorily. |  | |
| 2 | I understand that my participation is voluntary and that I am free to withdraw at any time without giving any reason, without my medical care or legal rights being affected. |  | |
| 3 | I understand that relevant data collected during the study may be looked at by individuals from the sponsor of the trial (Royal Free London NHS Foundation Trust) and responsible persons authorised by the sponsor, from regulatory authorities or from the NHS Trust, where it is relevant to my taking part in this research. I give permission for these individuals to have access to my study records |  | |
| 4 | I understand that the information collected about me will be used to support other research in the future and may be shared anonymously with other researchers. |  | |
| 5 | I agree to take part in the above study. |  | |
|  |  | **Please initial Yes or No** | |
|  |  | **Yes** | **No** |
| 6 | I wish to receive a copy of the study report and consent for my personal information to be stored in order to be contacted for this purpose. |  |  |

| Name of Participant | Date | Signature |
| --- | --- | --- |
| Name of person taking consent | Date | Signature |
