# Supplementary material for: Clinical and cost-effectiveness of ‘Live Well with Parkinson’s’ self-management intervention versus treatment as usual for improving quality of life for people with Parkinson’s: study protocol for a randomised controlled trial
Source: Trials. 2023 Dec 5;24:793. doi: 10.1186/s13063-023-07700-7 (PMC10698895; doi:10.1186/s13063-023-07700-7)
Supplement: Supplementary file 4 — Additional file 4. [file 13063_2023_7700_MOESM4_ESM.zip › Anonymised Participant Consent Form_Supp 2R1.doc]

Study Number:

**Participant Consent Form**

**(Randomised Controlled Trial)**

| **Name of Lead Researchers:** [Add name here]  **Name of Site Principal investigator**: [Add name here] | | **Please initial** |
| --- | --- | --- |
| 1 | I confirm that I have read and understood the information sheet dated............................... (version............) for the above study. I have had the opportunity to consider the information, ask questions and have had these answered satisfactorily. |  |
| 2 | I understand that my participation is voluntary and that I am free to withdraw at any time without giving any reason, without my medical care or legal rights being affected. |  |
| 3 | I understand that relevant sections of my medical notes and data collected during the study, may be looked at by individuals from the sponsor of the trial (Royal Free London NHS Foundation Trust) and responsible persons authorised by the sponsor, from regulatory authorities or from the NHS Trust, where it is relevant to my taking part in this research. I give permission for these individuals to have access to my records. |  |
| 4 | I understand that the information collected about me will be used to support other research in the future and may be shared anonymously with other researchers. |  |
| 5 | I understand that the sessions, for those in the intervention group, will be video/audio-recorded and transcribed (written out). I understand the recording(s) will be used for the purpose of making sure all data is correct and measures are scored as accurately as possible. I understand that the recordings will be kept securely and that no images of me will appear anywhere without my permission. All data will be kept confidential and made anonymous. |  |
| 6 | I understand that if during the study I lose capacity, I will be withdrawn from the study and my data collected up until that point retained by the study team unless requested otherwise. |  |
| 7 | I agree to take part in the above study. |  |

|  | **Please read the following questions carefully and initial yes or no for these optional parts of the study.** | | | | | **Yes** | **No** |  |
| --- | --- | --- | --- | --- | --- | --- | --- | --- |
| 8 | I agree to the assessment called the Unified Parkinson’s Disease Rating Scale (MDS-UPDRS) being video recorded. I understand the recording will only be used for the purpose of making sure that the measure is scored as accurately as possible. I understand that the recordings will be kept securely and that no images of me will appear anywhere without my permission. | | | | |  |  |  |
| 9 | I agree to my General Practitioner being informed of my participation in the study and including any necessary exchange of information about me between my GP and the research team | | | | |  |  |  |
| 10 | I give permission for the researchers to speak to my relative/carer | | | | |  |  |  |
| 11 | I am willing to donate a saliva sample for genetic analysis in Parkinson’s studies that will be stored until used in future research studies. I understand that this sample will be stored and treated in line with the rules by the Human Tissue Authority, to be analysed and used by the research team and its collaborators in future studies that are approved by a Research Ethics Committee both within and outside the UK. I understand that I will not receive feedback about findings from the sample. | | | | |  |  |  |
| 12 | I am willing to wear a motion sensor for 7 days on my wrist and/or trunk. I understand that I will not receive feedback about findings from wearing the sensor. | | | | |  |  |  |
| 13 | I am willing to be contacted in the future by the trial investigators for further information, for information of new studies which I have the option to consent to participate | | | | |  |  |  |
| 14 | I wish to receive a copy of the study report and consent for my personal information to be stored in order to be contacted for this purpose. | | | | |  |  |  |
|  | |  |  |  |  | | | |
| Name of participant | |  | Date |  | Signature | | | |
| Name of researcher taking consent | |  | Date |  | Signature | | | |
